# Bacteriological Assessment of Healthcare-Associated Pneumonia Using a Clone Library Analysis

**DOI:** 10.1371/journal.pone.0124697

**Published:** 2015-04-15

**Authors:** Shingo Noguchi, Hiroshi Mukae, Toshinori Kawanami, Kei Yamasaki, Kazumasa Fukuda, Kentarou Akata, Hiroshi Ishimoto, Hatsumi Taniguchi, Kazuhiro Yatera

**Affiliations:** 1 Department of Respiratory Medicine, University of Occupational and Environmental Health, Japan, Kitakyusyu, Fukuoka, Japan; 2 Department of Microbiology, University of Occupational and Environmental Health, Japan, Kitakyusyu, Fukuoka, Japan; Yale University, UNITED STATES

## Abstract

**Background:**

The causative pathogens of healthcare-associated pneumonia (HCAP) remain controversial, and the use of conventional cultivation of sputum samples is occasionally inappropriate due to the potential for oral bacterial contamination. It is also sometimes difficult to determine whether methicillin-resistant *Staphylococcus aureus* (MRSA) is a true causative pathogen of HCAP.

**Methods:**

We evaluated the bacterial diversity in bronchoalveolar lavage fluid (BALF) using molecular and cultivation methods in 82 HCAP patients. BALF specimens were obtained from the lesions of pneumonia using bronchoscopy. The bacterial flora was analyzed according to the clone library method using amplified fragments of the 16S ribosomal RNA gene with universal primers. In addition, sputum cultures and the above specimens were assessed.

**Results:**

Eighty (97.6%) of the 82 BALF samples obtained from the patients with HCAP showed positive polymerase chain reaction results. The predominant phylotypes detected in the BALF in this study included bacteria common in cases of community- and hospital-acquired pneumonia. In addition, the phylotypes of streptococci and anaerobes were detected in 19 (23.2%) and 8 (9.8%) cases, respectively. In particular, phylotypes of streptococci were highly detected among the patients 75 of age or older. *Staphylococcus aureus* was cultured in 23 (28.0%) cases using conventional cultivation methods and detected in only 6 (7.3%) cases as predominant phylotypes according to the clone library method.

**Conclusions:**

The clone library analysis of BALF in the HCAP patients detected heterogeneous bacteria and a high incidence of streptococci compared with that observed using cultivation methods. In addition, the results of our study may indicate a lower incidence of MRSA than previously expected in HCAP patients.

## Introduction

Healthcare-associated pneumonia (HCAP) is a category of respiratory infection that was recently documented in the 2005 American Thoracic Society (ATS)/Infectious Diseases Society of America (IDSA) guidelines [1]. The mortality rate of HCAP has been reported to be 20%, which is approximately twice that of community-acquired pneumonia (CAP) [2–5]. Recent reports have shown that 17.3–38.0% of patients with pneumonia can be categorized as having HCAP [3–7], and the number of patients with HCAP is expected to increase in association with the aging of the population [8].

Sputum examinations are widely used common methods for evaluating the causative pathogens of bacterial pneumonia. Due to unavoidable contamination with the upper respiratory tract, expectorated sputum samples are occasionally inadequate for identifying causative pathogens [9]. The increase in the number of elderly patients may also lead to an increase in the rate of etiologically unknown pathogens in pneumonia, as Cillóniz *et al*. reported that the rate of unknown pathogens increases with age (65–74 y: 56.3%, 75–84 y: 59.3%, ≥85 y: 68.6%) [10]. Quantitatively cultivating samples obtained directly from affected lesions via bronchoalveolar lavage or protected specimen brushing with bronchoscopy is more precise in terms of evaluating causative pathogens [1, 11].

Moreover, as aspiration is a major cause of pneumonia in elderly subjects, it is often difficult to determine whether cultured oral bacteria, including anaerobes and streptococci, which are often considered causative pathogens of aspiration pneumonia, are indeed true causative pathogens in conventional cultivation methods [12]. In fact, it has been reported that these oral bacteria are underestimated causative pathogens of pneumonia in the clinical setting [12, 13].

HCAP affects more elderly patients, who are unable to expectorate sputum and are prone to aspiration, than CAP. The causative pathogens of HCAP are therefore more controversial than those of CAP. According to previous reports, 32.5–67.9% of HCAP are etiologically unknown [4, 6, 7, 14]. With respect to causative pathogens, it has been reported that the causative pathogens of HCAP can be distinguished from those of CAP based on the higher rate of multidrug-resistant (MDR) pathogens, including methicillin-resistant *Staphylococcus aureus* (MRSA) [5, 15]. On the other hand, some other reports have documented no significant differences in causative pathogens between patients with HCAP and elderly patients with CAP [7, 16, 17]. Therefore, it may be insufficient to evaluate the causative pathogens of HCAP using conventional cultivation methods alone.

Molecular analyses, particularly sequence-based approaches using the 16S ribosomal RNA (rRNA) gene, have been reported to be cultivation-independent methods [18–22], and we recently reported the evaluation of causative pathogens in two types of respiratory infections, CAP [23] and bacterial pleurisy [24], using specimens obtained from bronchoalveolar lavage fluid (BALF) and pleural effusion, respectively. The results of our studies indicated the importance of oral bacteria, including streptococci and anaerobic pathogens [23, 24].

In the present study, we investigated bacterial diversities in patients with HCAP according to the clone library method using the 16S rRNA gene in BALF in comparison with the results obtained with conventional cultivation methods.

## Materials and Methods

### Study Population

This study was prospectively performed to recruit outpatients diagnosed with pneumonia at the University of Occupational and Environmental Health, Japan and referred hospitals (Wakamatsu Hospital of the University of Occupational and Environmental Health, Japan, Kyusyu Rosai Hospital, and Yamaguchi-ken Saiseikai Shimonoseki General Hospital) between April 2010 and November 2013. Patients with the following conditions were excluded: CAP, severe hypoxemia (requiring oxygenation at a rate of more than 5 L/min except for patients treated with intratracheal intubation and mechanical ventilation), severe cardiac dysfunction, shock, a poor general condition and lack of informed consent. The study was approved by the Human and Animal Ethics Review Committee of the University of Occupational and Environmental Health, Japan (No.09-118). All patients provided their written informed consent. The following information regarding was collected: age, sex, comorbid diseases, clinical manifestations and laboratory and radiological findings.

### Definitions

All patients were hospitalized and exhibited the presence of new areas of infiltration on chest radiographs and new clinical findings, including at least two of the following: fever, sputum production, coughing and leukocytosis (white blood cell count ≥ 10,000/μl). HCAP was defined according to the ATS/IDSA guidelines [1], including at least one of the following criteria: (1) hospitalization for two days or more within the preceding 90 days; (2) residence in a nursing home or extended care facility; (3) home infusion therapy (including antibiotics); (4) chronic dialysis within 30 days.

### Sample Collection

Fiberoptic bronchoscopy was performed and BALF specimens were then obtained from side of the lung where pulmonary infiltrates were identified on chest CT using 40 ml of sterile saline as previously described [23]. In addition, sputum samples were evaluated in patients with sputum production.

### Total Bacterial Cell Count and Cell Lysis Efficiency Analysis

We evaluated the total bacterial cell count and efficiency of cell lysis using epifluorescent microscopy as previously described [23, 24].

### Microbiological Evaluation using Conventional Cultivation Methods

Pathogens in the BALF and sputum samples were cultured using a semiquantitative method [23, 24]. Positive bacterial culture results for the respiratory tract were described as microbial identification. And all bacterial species were recorded when more than two bacterial species were cultured as microbial identification. The positive results of more than one for BALF and/or sputum culture, serological assessment and/or urinary antigens were described as “All” in the column of the table for microbial identification, while positive bacterial culture results for BALF and sputum were presented in the columns in the table, respectively. Serologic methods using single or paired sera were used to examine the presence of antibodies against *Mycoplasma pneumoniae* Complement Fixation Antigen (Denka Seiken, Tokyo, Japan), and a four-fold increase in the antibody titer between the paired sera was considered to be presumptive. In addition, the level of anti-*Chlamydophila pneumoniae* antibodies was determined using SeroCP ELISA for immunoglobulin G (IgG) and IgA (Savyon and Hain Lifescience, Nehren, Germany), and an increase of 1.0 in the index value for IgA and 1.35 in the index value for IgG between the paired sera was considered to be presumptive [25]. Urinary antigen tests were also performed to detect *Streptococcus pneumoniae* and *Legionella pneumophila* serogroup Ⅰ (Binax, Portland, ME, USA).

### DNA Extraction and Polymerase Chain Reaction Conditions

DNA samples were extracted from the BALF specimens by vigorously shaking the specimens with sodium dodecyl sulfate (final concentration: 3.0%) and glass beads. The 16S rRNA gene was subsequently amplified using a polymerase chain reaction (PCR) thermocycler (GeneAmp PCR system 9700; Applied Biosystems; Foster City, CA), as previously described [23, 24, 26].

### Clone Library Construction and Determination of Nucleotide Sequences

The PCR products were then cloned using a TOPO TA cloning kit (Invitrogen; Carlsbad, CA), and a total of 96 colonies were randomly selected from each clone library for the sequencing analysis. The nucleic acid sequences were determined on a 3130xl Genetic Analyzer (Applied Biosystems), as previously described [23, 24, 26].

### Homology Search

Highly accurate sequences selected by Phred quality values were trimmed from the primer and vector regions. Only the sequences having good quality were used for analyses. The remaining sequences were compared with an in-house database containing the 16S rRNA gene sequences of 5,870 type strains using the basic local alignment search tool algorithm. The 16S rRNA gene sequences of type strains were obtained from DNA Data Bank of Japan (http://WWWddbj.nig.ac.jp/) and the Ribosomal Database Project (http://rdp.cme.msu.edu/) [23, 24, 27].

### Definition of mono- or mixed-bacteria groups

We defined the “mono-bacteria-dominant group” as including patients in whom the predominant phylotype comprised over 80% of the detected bacterial phylotypes using the clone library method; the remaining patients were assigned to the “mixed-bacteria group.”

### Statistical Analysis

The statistical analyses were performed using the SPSS software package (version 19), and a value of P < 0.05 was considered to be statistically significant. For the statistical analyses, Fisher’s exact test for tables (2×2) and the Mann-Whitney (non-parametric) test were used.

## Results

### Patient Characteristics

**Fig 1:**
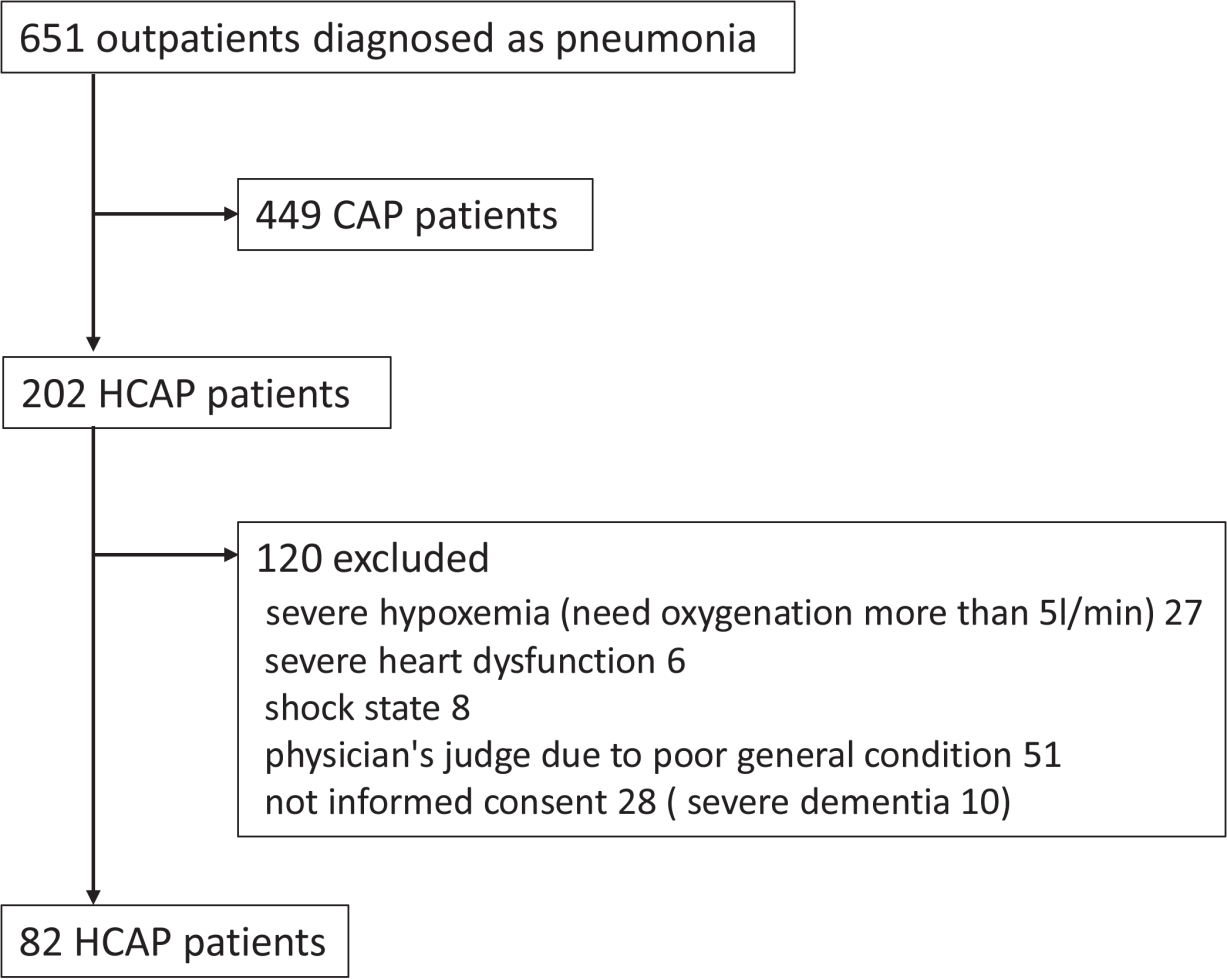
Patient inclusion and exclusion flow diagram.

A total of 651 outpatients diagnosed with pneumonia were assessed for eligibility, and 82 HCAP patients who underwent bronchoscopic examinations were ultimately evaluated in this study (Fig 1). In the background characteristics of the 82 patients, 52 patients (63.4%) had been hospitalized for two days within the preceding 90 days, 29 patients (35.4%) had been in residence at a nursing home or extended care facility and 19 patients (23.2%) had been receiving home infusion therapy (including antibiotics). None of the subjects had received chronic dialysis within the previous 30 days. The baseline characteristics of the patients are presented in Table 1.

**Table 1:** Clinical and laboratory features of the 82 patients with HCAP.

Age (y); mean ± SD	75.5 ± 10.3		Radiographic findings				
Sex (male / female)		57 / 25			Bilateral lung involvement	42	(51.2)
BMI; mean ± SD^§1^		20.3 ± 4.0			Pleural effusion	24	(29.3)
							
Comorbidity diseases				Previous antibiotic treatment		13	(15.9)
	Neoplastic disease	25	(30.5)	Use of antibiotics within the previous 90d		28	(34.1)
	Chronic pulmonary disease	37	(45.1)	Smoking history		34	(41.5)
	Cerebrovascular disease	29	(35.4)	Alcohol history		19	(23.2)
	Chronic cardiac disease	16	(19.5)	Performance status^§4^			
	Chronic liver disease	7	(8.5)		0	10	(12.2)
	Chronic renal disease	9	(11.0)		1	30	(36.6)
	Diabetes mellitus	16	(19.5)		2	13	(15.9)
	Collagen disease	9	(11.0)		3	11	(13.4)
	No comorbidity disease	0	(0.0)		4	18	(22.0)
	Two or more comorbidities	56	(68.3)				
				Mechanical ventilation		13	(15.9)
Clinical parameters							
	Orientation disturbance (confusion)	23	(28.0)	Stratification by PSI			
	Systolic BP < 90 mm Hg or diastolic	20	(24.4)		Ⅰ-Ⅲ	15	(18.3)
	BP ≤ 60 mm Hg				ⅠV	45	(54.9)
	Pulse rate ≥ 125 beats/min	9	(11.0)		V	22	(26.8)
	Respiratory rate ≥ 30 /min ^§2^	10	(14.7)				
	SpO_2_ ≤ 90%, PaO_2_ ≤ 60 mm Hg	44	(53.7)	In hospital mortality		7	(8.5)
					Ⅰ-Ⅲ	0	(0.0)
Laboratory findings					ⅠV	3	(3.7)
	pH < 7.35^§3^	7	(13.7)		V	4	(4.9)
	BUN ≥ 21mg/dl	36	(43.9)				
	Na < 130 mEq/ml	10	(12.2)				
	Glucose ≥ 250mg/dl	2	(2.4)				
	Hematocrit < 30%	16	(19.5)				
	Albumin < 3.0g/dl	34	(41.5)				

Data are presented as n (%) or mean ± SD unless otherwise stated.

*Definition of abbreviations*: HCAP, healthcare-associated pneumonia; BMI, body mass index; BP, blood pressure; SpO_2_, pulse oximetric saturation; PaO_2_, partial pressure of arterial oxygen; BUN, blood urea nitrogen; PSI, pneumonia severity index; SD, standard deviation

^§1^BMI was evaluated in 59 patients

^§2^Respiratory rate was evaluated in 68 patients

^§3^Arterial blood gas analysis was performed in 51 patients

^§4^ 0, can be active without any problems or limitations, daily life the same as before the onset; 1, intense activity limited, but can walk and perform light work or work while sitting; 2, can walk and perform all personal care, but cannot work; more than 50% of daytime hours out of bed; 3, can only do limited personal care; more than 50% of daytime hours spent in bed or chair; 4, cannot move at all or perform personal care, all day spent in bed or chair

### Total Bacterial Cell Numbers

Eighty of the 82 (97.6%) BALF specimens showed positive PCR results for the 16S rRNA gene according to the clone library method. The bacterial numbers according to the epifluorescent microscopic analysis among the 80 patients positive for 16S rRNA on PCR in the BALF samples ranged from 1.2×10^4^ to 8.3×10^8^ cells/mL (median, 3.3×10^7^ cells/mL). In addition, the total cell number in the two cases with negative results on PCR for the 16S rRNA gene was less than 10^4^ cells/mL. The efficiency of cell lysis was maintained at 80% or greater in all samples.

### Comparison Between the Results of the Conventional Cultivation Methods and the Predominant Bacterial Phylotypes Determined According to the Clone Library Method

The predominant phylotypes in the BALF samples detected according to the clone library method and the results identified using the conventional cultivation methods are shown in Table 2. The clone library method demonstrated a positive PCR rate of 97.6% in the BALF samples, in contrast, microbes were identified in 69 of the 82 (84.1%) cases according to conventional cultivation methods. In addition, microbes were identified in 59 (72.0%) and 43 (52.4%) of the 82 cases in the BALF and sputum specimens, respectively. According to the clone library method, streptococci (23.2%) were the most frequently detected phylotypes, followed by *H*. *influenzae* (17.1%), *S*. *pneumoniae* (11.0%), anaerobes (*Prevotella* species, *Fusobacterium* species, *Parvimonas* species, *Veillonella* species, *Porphyromonas* species) (9.8%), *P*. *aeruginosa* (9.8%) and *S*. *aureus* (7.3%). On the other hand, *S*. *aureus* (29.3%) was isolated most frequently, followed by *P*. *aeruginosa* (19.5%), *S*. *pneumoniae* (12.2%), streptococci (12.2%), *Klebsiella* species (12.2%) and *H*. *influenzae* (11.0%) according to the conventional cultivation methods. The rate of concordance between the results of the clone library method and conventional cultivation method was >90% for *S*. *pneumoniae* and *H*. *influenzae*, 30–50% for streptococci, *Corynebacterium* species, *Moraxella catarrhalis*, *Klebsiella* species, *P*. *aeruginosa* and *E*. *coli*, and <10% for *S*. *aureus*. In addition, the results of the clone library method in the 13 cases in which the bacteria were not cultured according to conventional cultivation methods were as follows: *S*. *aureus* 1, streptococci 3, *Gemella* species 1, *H*. *influenzae* 3, *P*. *aeruginosa* 1, anaerobes 3, and *M*. *pneumoniae* 1. *S*. *pneumoniae* and *H*. *influenzae*, which are known to be common pathogens of CAP, were frequently identified using both the clone library method and conventional cultivation methods. *S*. *aureus* and *P*. *aeruginosa*, which are known to be common pathogens of HAP, were also identified using both methods; however, the results of the clone library method showed rates of the phylotypes of *S*. *aureus* and *P*. *aeruginosa* that were less than half of those obtained using the conventional cultivation methods. On the other hand, streptococci and anaerobes were more frequently detected using the clone library method than conventional cultivation methods. Ten of the 82 patients exhibited positive bacterial cultures for the sputum samples in association with negative results for the BALF samples. The bacterial species cultured using sputum samples in these 10 cases were as follows: *S*. *pneumonia* 2, MSSA 1, MRSA 1, *Klebsiella* spp. 1, oral bacteria 2, MSSA+*E*. *coli* 2, and MSSA+*P*. *aeruginosa* 1. Supplemental information regarding the detailed results of each case is provided in the S1 Table.

**Table 2:** Predominant bacteria according to the molecular method and conventional cultivation methods.

			Clone Library Method in BALF		Conventional Cultivation Methods					
			Case detected as the predominant phylotype		All^§^		BALF		Sputum	
Gram-positive pathogens										
	*Streptococcus pneumoniae* ^#^		9	(11.0)	10	(12.2)	8	(9.8)	4	(4.9)
	*Staphylococcus aureus*		6	(7.3)	23	(28.0)	16	(19.5)	16	(19.5)
	Methicillin-susceptible *S*. *aureus*				11	(13.4)	6	(7.3)	7	(8.5)
	Methicillin-resistant *S*. *aureus*				12	(14.6)	9	(11.0)	8	(9.8)
	*Staphylococcus* species				1	(1.2)	1	(1.2)		
	*Streptococcus* species (except *S*. *pneumoniae*)		19	(23.2)	10	(12.2)	9	(11.0)	6	(7.3)
	*Streptococcus anginosus* species		4	(4.9)	1	(1.2)	1	(1.2)	1	(1.2)
	*Streptococcus* species (except *S*. *pneumoniae*, *S*. *anginosus* species)		15	(18.3)	9	(11.0)	8	(9.8)	5	(6.1)
	*Corynebacterium* species		4	(4.9)	5	(6.1)	4	(4.9)	1	(1.2)
	*Gemella* species		1	(1.2)						
Gram-negative pathogens										
	*Haemophilus influenzae*		14	(17.1)	9	(11.0)	8	(9.8)	3	(3.7)
	*Moraxella catarrhalis*		1	(1.2)	2	(2.4)	2	(2.4)	1	(1.2)
	*Klebsiella* species		3	(3.7)	10	(12.2)	7	(8.5)	6	(7.3)
	*Pseudomonas aeruginosa*		8	(9.8)	16	(19.5)	14	(17.1)	8	(9.8)
	*Pseudomonas* species (except *P*. *aeruginosa*)				1	(1.2)			1	(1.2)
	*Escherichia coli*		2	(2.4)	3	(3.7)	2	(2.4)	1	(1.2)
	*Enterobacter* species		1	(1.2)	2	(2.4)	1	(1.2)	1	(1.2)
	*Acinetobacter* species				2	(2.4)	2	(2.4)		
	*Citrobacter* species				1	(1.2)	1	(1.2)		
	*Serratia* species				1	(1.2)	1	(1.2)		
	*Neisseria* species		2	(2.4)						
Anaerobic pathogens										
	*Prevotella* species		3	(3.7)						
	*Fusobacterium* species		1	(1.2)						
	*Parvimonas* species		2	(2.4)						
	*Veillonella* species		1	(1.2)						
	*Porphyromonas* species		1	(1.2)						
Atypical pathogens										
	*Mycoplasma pneumoniae*		1	(1.2)						
*Nocardia* species			1	(1.2)	1	(1.2)	1	(1.2)	1	(1.2)
Oral bacteria					7	(8.5)	6	(7.3)	4	(4.9)
No pathogen identified			2	(2.4)	13	(15.9)	23	(28.0)	39	(47.6)
Total isolates			-		105		83		53	

Data are presented as n (%) unless otherwise stated. Percentage refer to the total number of patients (n = 82)

*Definition of abbreviations*: HCAP, healthcare-associated pneumonia; BALF, bronchoalveolar lavage fluid

^§^Including positive results of BALF and/or sputum culture, serological assessment, and urinary antigens

^#^The results of urinary antigen tests to detect *Streptococcus pneumoniae* were positive in 4 patients

### Evaluation of the Proportion of Microbiota According to the Clone Library Method

30 patients (37.5%) were categorized as belonging to the monobacteria-dominant group (S1A Fig), while 50 patients (62.5%) were classified as belonging to the mixed bacteria group (S1B Fig). In the mixed bacteria group, the phylotypes of streptococci were most frequently detected in 31 of 50 (62.0%) cases, followed by those of anaerobes (46.0%), *Corynebacterium* species (18.0%), *S*. *aureus* (16.0%), *Gemella* species (14.0%) and *P*. *aeruginosa* (12.0%). In contrast, more than two pathogens were identified in 28 of 80 (35.0%) cases using conventional cultivation methods, and *S*. *aureus* and/or *P*. *aeruginosa* were identified in 20 of these 28 (71.4%) cases.

**Fig 2:**
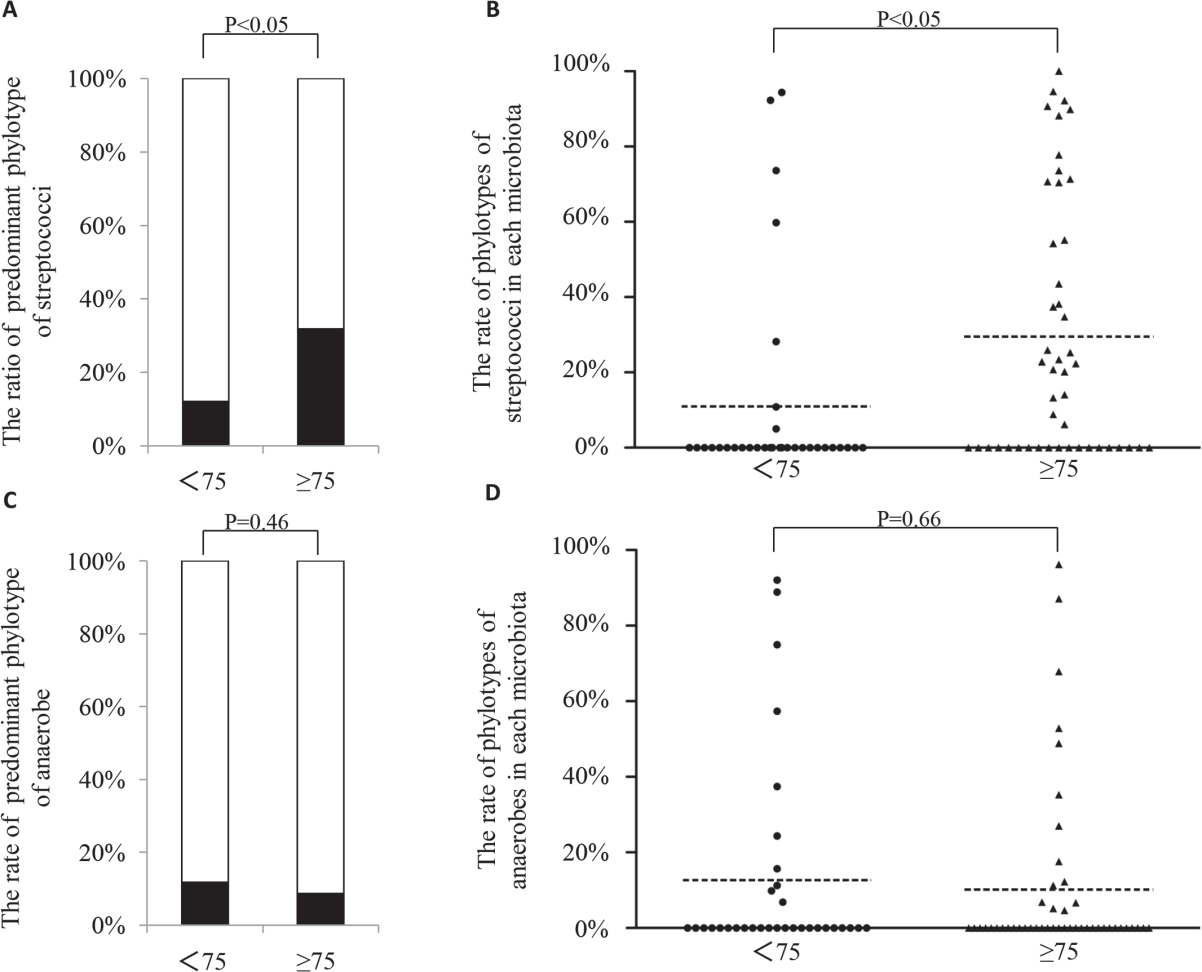
Comparison of the predominat phylotype and the rates of phylotypes of streptococci or anaerobes. A) With respect to streptococci, the rate of a predominant phylotype was significantly higher in the patients equal to or older than 75 years of age (the rates of streptococci in the patients younger than 75 years old and equal to or older than 75 years of age were 12.1% and 31.9%, respectively; P<0.05); B) The rates of the phylotypes in each microbiota were significantly higher in the patients equal to or older than 75 years of age (<75 vs ≥75 were 11.0 ± 27.0% and 29.5 ± 34.1%, respectively; P<0.05); C-D) Regarding anaerobes, the rate of the predominant phylotype (<75 vs ≥75 were 11.8% vs 8.7%, respectively; P = 0.46) and the phylotypes in each microbiota (<75 vs ≥75 were 12.7 ± 26.5% vs 10.2 ± 23.1%, respectively; P = 0.66) were not significantly different between the two age groups.

We divided the HCAP patients into two age groups (younger than 75 years of age and equal to or older than 75 years of age). There were no significant differences in the rates of the predominant phylotypes between these two age groups (P = 0.29). With respect to streptococci, the rates of the predominant phylotype of streptococci and the phylotypes of streptococci in each microbiota among the patients 75 years of age or older were significantly higher than those observed among the patients less than 75 years of age (Fig 2A and 2B). In contrast, the rates of anaerobes were not significantly different between the two age groups (Fig 2C and 2D).

### Comparison of the Results of the Conventional Cultivation Method for Assessing Sputum with the Clone Library Method

We compared the results of the phylotypes in BALF specimens with the results of sputum cultivation in the cases of positive sputum bacterial cultivation (Table 3). With respect to *S*. *pneumoniae* and *H*. *influenzae*, the results for sputum cultivation were correlated with the first or second phylotypes in the results obtained using the clone library method. In contrast, the rate of concordance between the first or second phylotypes in the results obtained using the clone library method and sputum cultivation were 12.5%, 33.3% and 50.0% for *S*. *aureus*, *Klebsiella* species and *P*. *aeruginosa*, respectively. In particular, *S*. *aureus* exhibited a low rate of concordance, and 13 of 16 (81.3%) cases in which *S*. *aureus* was detected in the sputum cultivation showed a low proportion of *S*. *aureu*s (less than 5%) according to the clone library method.

**Table 3:** The difference of bacteria according to the molecular method in sputum cultivation.

		Sputum		Clone Library Method in BALF							
		The number detected in cultivation		The predominant phylotype		The second phylotype		Less than the third phylotype (except others^§^)		Others^§^	
Gram-positive pathogens											
	*Streptococcus pneumoniae*	4		3	(75.0)	1	(25.0)				
	*Staphylococcus aureus*	16		2	(12.5)			1	(6.3)	13	(81.3)
	*Streptococcus anginosus* species	1		1	(100)						
	*Streptococcus* species (except *S*. *pneumoniae*,	5		3	(60.0)					2	(40.0)
	*S*. *anginosus* species)										
	*Corynebacterium* species	1				1	(100)				
Gram-negative pathogens											
	*Haemophilus influenzae*	3		3	(100.0)						
	*Moraxella catarrhalis*	1		1	(100.0)						
	*Klebsiella* species	6		2	(33.3)					4	(66.7)
	*Pseudomonas aeruginosa*	8		3	(37.5)	1	(12.5)			4	(50.0)
	*Pseudomonas* species (except *P*. *aeruginosa*)	1								1	(100)
	*Escherichia coli*	1								1	(100)
	*Enterobacter* species	1		1	(100)						
*Nocardia* species		1		1	(100.0)						

*Definition of abbreviations*: BALF, bronchoalveolar lavage fluid

^§^The phylotypes that dominated less than 5% in each clone library were classified as "others"

### Results of the Clone Library Method and Effects of Treatment in the Cases in which MRSA was Isolated using Conventional Cultivation Methods

We evaluated the results of the conventional cultivation methods and treatments for *S*. *aureus* (MRSA), *P*. *aeruginosa* and *Klebsiella* species due to the discrepancies between the results of the clone library method and conventional cultivation methods. The characteristics of the 12 cases in which MRSA was isolated using conventional cultivation of the sputum and/or BALF samples are shown in Table 4. According to the clone library method, only 3 of these 12 (25.0%) cases involved more than 5% clones of *S*. *aureus*. In addition, 9 of the 12 (75.0%) patients demonstrated a clinical improvement in pneumonia following treatment with antibiotics other than anti-MRSA drugs, while the remaining three patients (No. 1, 7, and 9) were successfully treated with anti-MRSA agents. Two (No. 7 and 9) of these three cases were considered to involve mixed infections, as the rate of *S*. *aureus* in the microbiota of the BALF was greater than 5%, according to the clone library method. In addition, we evaluated *P*. *aeruginosa* and *Klebsiella* species; however, we were unable to assess the differences between the results of the clone library method and the conventional cultivation methods for these species because most of the patients were treated with broad-spectrum antibiotics covering *P*. *aeruginosa* and *Klebsiella* (S2 and S3 Tables).

**Table 4:** Results of the molecular method and antibiotics efficacy in patients with positive cultivation of MRSA.

No.^§^	Cultivation		The results of Clone Library Method of 16S ribosomal RNA gene		Effective antibiotics
	Sputum	BALF	BALF		
			Predominant phylotype (%, Clones/clones)	Proportion of *S*. *aureus (%*, *Clones/clones)*	
1	MRSA,	MRSA,	*Streptococcus salivarius*		MEPM→ TAZ/PIPC+VCM
	*Pseudomonas aeruginosa*	*Pseudomonas aeruginosa*	43.0% (34/79)	1.3% (1/79)	
2	MRSA,	MSSA,	*Streptococcus pneumoniae*		SBT/ABPC
	*Streptococcus pneumoniae*	*Streptococcus pneumoniae*	100% (83/83)	0% (0/83)	
3	MRSA	MRSA	*Streptococcus salivarius*		LVFX
			81.6% (62/76)	0% (0/76)	
4	MRSA	MRSA	*Streptococcus oralis*		TAZ/PIPC
			73.6% (64/87)	2.3% (2/87)	
5	MRSA	No growth	*Neisseria mucosa*		MEPM
			55.0% (33/60)	0% (0/60)	
6	MRSA,	MSSA	*Staphylococcus aureus*		TAZ/PIPC
	*Pseudomonas aeruginosa*		54.7% (52/95)	54.7% (52/95)	
7	MRSA,	MRSA,	*Streptococcus oralis*		LVFX
	*Streptococcus* species	*Streptococcus* species,	43.5% (40/92)	12.0% (11/92)	→LZD
		*Corynebacterium* species			
8	MRSA,	MRSA,	*Streptococcus intermedius*		TAZ/PIPC
	*Streptococcus* species	*Streptococcus* species,	27.2% (25/92)	0% (0/92)	
		*Klebsiella pneumoniae*			
9	N.A	MRSA	*Corynebacterium simulans*		MEPM+LZD
			54.8% (46/84)	23.8% (20/84)	
10	N.A	MRSA,	*Streptococcus oralis*		SBT/ABPC
		*Streptococcus* species,	70.7% (53/75)	0% (0/75)	
		*Pseudomonas aeruginosa*			
11	N.A	MRSA,	*Pseudomonas aeruginosa*		TAZ/PIPC
		*Pseudomonas*. *aeruginosa*	97.4% (76/78)	0% (0/78)	
12	N.A	MRSA,	*Corynebacterium simulans*		SBT/ABPC
		*Corynebacterium* species	58.9% (53/90)	3.3% (3/90)	

*Definition of abbreviation*: MRSA, methicillin-resistant *staphylococcus aureus*; MSSA, methicillin-susceptible *staphylococcus aureus*; BALF, bronchoalveolar lavage fluid; SBT/ABPC, ampicillin/sulbactam; TAZ/PIPC, piperacillin/tazobactam; MEPM, meropenem; LVFX, levofloxacin; VCM, vancomycin; LZD, linezolid; N.A, not analyzed

^§^Case numbers were as follow: No.1, case35; No.2, case38; No.3, case39; No.4, case40; No.5, case44; No.6, case60; No.7, case66; No.8, case67; No.9, case31; No.10, case57; No.11, case63; No.12, case77

## Discussion

In the present study, we analyzed BALF specimens obtained from 82 HCAP patients using the clone library method in order to assess the bacterial 16S rRNA gene in comparison with the results of conventional cultivation methods. To the best of our knowledge, this is the first report to evaluate the microbiota of affected lung lesions in HCAP patients using BALF specimens. The results of this study suggest that streptococci and anaerobes play an important role in the pathogenesis of HCAP in addition to common pathogens, such as *S*. *pneumoniae* and *H*. *influenzae*. In addition, the results of the cultivation methods occasionally did not correctly reflect the microbiota of the lesions of pneumonia, particularly MRSA; thus, our results provide additional bacterial information with respect to HCAP.

We previously reported the importance of streptococci and anaerobes in patients with CAP [23] and bacterial pleurisy [24] using this molecular method and believe that our clonal microflora analysis is more effective method for detecting the possible cause. Most previous reports investigating causative pathogens in HCAP patients have been conducted using sputum cultivation-based methods; however, it remains controversial whether the cultured bacteria isolated from sputum samples are indeed causative pathogens [11, 28]. In the present study, we evaluated BALF samples obtained from affected lung lesions via bronchoscopy in order to strictly evaluate the contribution of bacteria, particularly oral streptococci and anaerobes, to the development of pneumonia, compared with the findings of sputum cultivation, which are easily contaminated due to contact with the oral cavity.

The predominant phylotypes detected using the clone library method in the HCAP patients showed obvious differences compared to our previous results obtained using the clone library method in patients with CAP. In our former report of CAP using the clone library method, *S*. *pneumoniae*, *H*. *influenzae* and *M*. *pneumoniae* occupied 35 of 64 (54.7%) cases [23]. In contrast, only 24 of 82 (29.3%) HCAP patients presently evaluated exhibited these three common pathogens, and the clone library method revealed *S*. *aureus* and *P*. *aeruginosa* in 14 cases (17.1%) among the HCAP patients compared to two cases (3.1%) among the CAP patients [23]. Therefore, the possible causative bacteria of HCAP included species found in both CAP and HAP patients, as well as those previously reported by Shindo *et al*. [14] and Attridge *et al*. [29]. Meanwhile, *H*. *influenzae* was frequently detected compared with the findings of previous reports (2.8–11.9%) [4–6, 14]. *H*. *influenzae* is often found in patients with pneumonia accompanied with comorbidities [10], and our results confirmed that *H*. *influenzae* is a major bacterial species in HCAP patients with comorbid diseases.

It has been reported that streptococci play important roles in the development of respiratory infections [12, 13, 30, 31] and have been identified to be causative pathogens in 5.0% to 14.1% of patients with HCAP [5, 14, 32, 33]. The incidence of streptococci (23.2%) observed as the predominant phylotype was higher in this study than that noted in our previous report of CAP patients (9.4%) [23]. We previously reported that a total bacterial cell count of more than 10^4^ cells/mL in BALF specimens is a useful criterion for diagnosing bacterial infection in BALF [23]; the cell counts of the BALF specimens, which exhibited high rates of streptococci, fulfilled this criterion in the present study. Therefore, we believe that streptococci play important roles in the microbiota in patients with pneumonia. Possible underestimation of streptococci as causative pathogens has also been previously reported by Shinzato *et al*. [13], and streptococci may play etiologically more important roles in the pathogenesis of pneumonia than previously believed, although these microbes are usually recognized to be a source of oral colonization. In relation to aspiration in elderly patients, Teramoto *et al*. [34] reported that aspiration often contributes to the pathogenesis of pneumonia, especially in patients over 70–80 years of age, El-Solh *et al*. [12] found that oral aerobic microorganisms are important pathogens in patients with aspiration pneumonia and Bousbia *et al*. reported that the bacterial flora in the BALF are polyclonal and that streptococci are associated with aspiration pneumonia detected according to the molecular method in patients who develop pneumonia in the intensive care unit [22]. Meanwhile, although the results showing the detection of streptococci in much older patients in this study may support the findings of the above report, the correlation between the presence of streptococci in the lower respiratory tract and the development of aspiration pneumonia must be evaluated in future studies.

In the current study, there were discrepancies in the rate of detection of *S*. *aureus*, *P*. *aeruginosa* and *K*. *pneumoniae* using the clone library method and conventional cultivation methods; in particular, the cultivation results and treatment efficacy were not in agreement in the cases in which MRSA was cultured. The clinical significance of MRSA as a causative pathogen in patients with HCAP remains controversial [35], and several reports have demonstrated that, even when MRSA are cultured using sputum samples, these bacteria can occasionally be incorrectly identified as causative pathogens [28, 36, 37]. Twenty-three (28.0%) of the 82 patients evaluated in this study were found to have *S*. *aureus* according to the cultivation methods, similar to the findings of previous reports [5, 15, 35]. In contrast, *S*. *aureus* was detected in only 6 (7.3%) cases according to the clone library method. Interestingly, 75% of the cases in which MRSA was isolated using the conventional cultivation methods were successfully treated without anti-MRSA antimicrobials. A recent report by Leone *et al*. also demonstrated a high negative predictive value for the pathogenic role of *S*. *aureus* using rapid diagnostic tests (real-time PCR) of BALF samples in patients with ventilator-associated pneumonia [38]. It has also been reported that pathogens such as MRSA can be easily cultured using respiratory samples, and it is necessary to consider that the use of home nursing care and prior hospitalization, which correspond to the content of the definition of HCAP, are risk factors for the detection of MRSA [39, 40]. Therefore, our results suggest that careful consideration might occasionally be necessary as to whether it is a possible cause or only an agent of colonization when it was cultured, although the clinical background and chest X-ray and/or CT image findings must be taken into consideration in order to select the proper antibiotics, including anti-MRSA drugs.

There are several limitations associated with the present study that should be kept in mind when interpreting the results [23, 24]. First, the universal primers used in this study could not be used to amplify all of the bacterial 16S rRNA genes, and the sensitivity of the primers was approximately 92% of the bacterial species registered in the Ribosomal Database Project II database. The remaining approximately 8% of bacteria undetectable with these primers include no reported human causative pathogens. Second, the number of clones analyzed in this study was approximately 100 per library, suggesting that this method may be unable to detect bacterial 16S rRNA gene sequences present at very small fractions (less than 1% of each sample).

## Conclusion

We herein evaluated the bacterial phylotypes in BALF specimens obtained from HCAP patients using the clone library method compared with the results of conventional cultivation methods. This study demonstrated that bacteria in the lower respiratory tract are extremely heterogeneous in patient with HCAP and that streptococci play a more important role than previously reported in this patient population. In addition, the results of the cultivation-independent method indicate that cultivation methods may not correctly reflect a possible cause, particularly MRSA, in HCAP patients, and further studies are needed to evaluate the potential of MRSA as a causative pathogen.

## Supporting Information

S1 Table Comparison of detected bacteria between conventional cultivation methods and the clone library method in the BALF. (DOCX)

S2 Table Results of the molecular method and antibiotics efficacy in patients with positive cultivation of *Pseudomonas aeruginosa*. (DOCX)

S3 Table Results of the molecular method and antibiotics efficacy in patients with positive cultivation of *Klebsiella pneumonia*. (DOCX)

S1 Fig Percentage of detected phylotypes in the “monobacteria-dominant” and “mixed bacteria” groups. A) Percentage of phylotypes in each sample among the 30 patients in the “monobacteria-dominant group”; B) Percentage of phylotypes in each sample among the 50 patients in the “mixed bacteria group.” Phylotypes present at a rate of less than 5% in each library were classified as “others.”
(DOCX)
