# Supplementary material for: Bacteriological Assessment of Healthcare-Associated Pneumonia Using a Clone Library Analysis
Source: PLoS One. 2015 Apr 15;10(4):e0124697. doi: 10.1371/journal.pone.0124697 (PMC4398420; doi:10.1371/journal.pone.0124697)
Supplement: S1 Table — (DOCX) [file pone.0124697.s001.docx]

Table S1. Comparison of detected bacteria between conventional cultivation methods and the clone library method in the BALF

| Case No. | Age /Sex | BALF | | | |  | Sputum | |
| --- | --- | --- | --- | --- | --- | --- | --- | --- |
|  |  | Cell number (cells/mL) | Gram stain | Cultivation | Clone Library Method of 16S ribosomal RNA gene |  | Gram stain | Cultivation |
|  |  |  |  |  | Predominant phylotype^§1^ |  |  |  |
|  |  |  |  |  | (Clones/clones, %) |  |  |  |
| 1 | 62 / M | 3.2×10^6^ | GPC | No growth | *Streptococcus oralis* |  | N.A | |
|  |  |  |  |  | 64/87, 73.6% |  |  |  |
| 2 | 71 / M | 3.2×10^6^ | (-) | *Streptococcus oralis* | *Parvimonas micra* |  | GPC | *Streptococcus oralis* |
|  |  |  |  |  | 34/63, 54.0% |  |  |  |
| 3 | 56 / M | 4.2×10^6^ | GNR | *Pseudomonas aeruginosa* | *Pseudomonas aeruginosa* |  | GPC, | *Pseudomonas aeruginosa* |
|  |  |  |  |  | 77/77, 100% |  | GPR, |  |
|  |  |  |  |  |  |  | GNR |  |
| 4 | 79 / F | 8.3×10^7^ | GPR | *Staphylococcus aureus* (MSSA) | *Haemophilus influenzae* |  | N.A | |
|  |  |  |  |  | 81/81, 100% |  |  |  |
| 5 | 66 / F | 1.3×10^6^ | (-) | *Streptococcus parasanguinis*, | *Pseudomonas aeruginosa* |  | GPC, | Oral bacteria |
|  |  |  |  |  | 45/81, 55.6% |  | GPR, |  |
|  |  |  |  | *Acinetobacter haemolyticus* |  |  | GNR |  |
| 6 | 77 / M | 1.5×10^6^ | (-) | No growth | *Staphylococcus aureus* |  | N.A | |
|  |  |  |  |  | 43/81, 53.1% |  |  |  |
| 7 | 80 / F | 1.6×10^7^ | (-) | No growth | *Staphylococcus aureus* |  | GPC | *Corynebacterium* species |
|  |  |  |  |  | 36/65, 55.4% |  |  |  |
| 8 | 81 / M | 1.9×10^6^ | GPC, | *Moraxella catarrhalis* | *Moraxella catarrhalis* |  | GPC, | *Moraxella catarrhalis* |
|  |  |  | GNC |  | 76/83, 91.6% |  | GNR |  |
| 9 | 60 / M | 6.9×10^6^ | GPC, | *Streptococcus pneumoniae* | *Streptococcus pneumoniae* |  | N.A | |
|  |  |  | GPR |  | 24/78, 30.8% |  |  |  |
| 10 | 65 / M | 1.0×10^7^ | GPC | No growth | *Parvimonas micra* |  | GPC | No growth |
|  |  |  |  |  | 28/76, 36.8% |  |  |  |
| 11 | 78 / M | 2.1×10^8^ | GPC | Oral bacteria | *Streptococcus salivarius* |  | GPC, | Oral bacteria |
|  |  |  |  |  | 37/63, 58.7% |  | GNR |  |
| 12 | 84 / M | 2.5×10^4^ | (-) | Oral bacteria | *Streptococcus oralis* |  | N.A | |
|  |  |  |  |  | 31/44, 70.5% |  |  |  |
| 13 | 84 / M | 9.7×10^7^ | GPC | *Staphylococcus aureus* (MSSA) | *Streptococcus intermedius* |  | N.A | |
|  |  |  |  |  | 83/90, 92.2% |  |  |  |
| 14 | 78 / F | 1.2×10^4^ | (-) | *Haemophilus influenza* | *Haemophilus influenzae* |  | GPC, | *Staphylococcus aureus* (MSSA) |
|  |  |  |  |  | 45/64, 70.3% |  | GPR |  |
| 15 | 68 / F | 3.7×10^4^ | GPC | *Streptococcus pneumoniae* ^#^ | *Escherichia coli* |  | (-) | *Streptococcus pneumoniae* |
|  |  |  |  |  | 46/82, 56.1% |  |  |  |
| 16 | 78 / M | 1.8×10^7^ | (-) | Oral bacteria | *Prevotella* species |  | N.A | |
|  |  |  |  |  | 41/70, 58.6% |  |  |  |
| 17 | 78 / M | 7.1×10^6^ | GPC | *Streptococcus pneumoniae*^#^ | *Streptococcus pneumoniae* |  | GPC, | *Staphylococcus aureus* (MSSA) |
|  |  |  |  |  | 90/90, 100% |  | GNC, |  |
|  |  |  |  |  |  |  | GNR |  |
| 18 | 80 / M | 3.1×10^4^ | (-) | No growth | *Prevotella* species |  | GPC, | No growth |
|  |  |  |  |  | 24/78, 30.8% |  | GNR |  |
| 19 | 60 / F | 3.4×10^6^ | GPC, | No growth | *Streptococcus parasanguinis* |  | N.A | |
|  |  |  | GPR |  | 46/82, 59.8% |  |  |  |
| 20 | 70 / M | 3.1×10^4^ | (-) | *Nocardia* species | *Nocardia exalbida* |  | GPR | *Nocardia* species |
|  |  |  |  |  | 48/68, 70.6% |  |  |  |
| 21 | 74 / M | 2.1×10^8^ | GPC, GNR | *Staphylococcus aureus* (MSSA),  *Pseudomonas aeruginosa* | *Staphylococcus aureus* |  | GPC, | *Staphylococcus aureus* (MSSA), |
|  |  |  |  |  | 83/87, 95.4% |  | GPR, |  |
|  |  |  |  |  |  |  | GNC, |  |
|  |  |  |  |  |  |  | GNR | *Pseudomonas aeruginosa* |
| 22 | 45 / F | 3.1×10^6^ | (-) | *Staphylococcus aureus* (MSSA) | *Haemophilus influenzae* |  | GNC, | *Staphylococcus aureus* (MSSA) |
|  |  |  |  |  | 83/88, 94.3% |  | GNR |  |
| 23 | 78 / F | 6.6×10^6^ | (-) | *Escherichia coli* | *Escherichia coli* |  | GPC, | No growth |
|  |  |  |  |  | 45/72, 62.5% |  | GNR |  |
| 24 | 75 / M | <10^4^ | (-) | No growth | (-) |  | GPC | *Staphylococcus aureus* (MSSA) |
| 25 | 81 / M | 1.2×10^6^ | GPC | No growth | *Mycoplasma pneumoniae* |  | N.A | |
|  |  |  |  |  | 73/84, 86.9% |  |  |  |
| 26 | 80 / M | 4.3×10^6^ | GPC | No growth | *Streptococcus pneumoniae* |  | GPC | *Streptococcus pneumoniae* |
|  |  |  |  |  | 81/81, 100% |  |  |  |
| 27 | 70 / M | 7.6×10^7^ | GPC, GPR, GNR | *Pseudomonas aeruginosa* | *Pseudomonas aeruginosa* |  | GPC, | *Pseudomonas aeruginosa* |
|  |  |  |  |  | 15/64, 23.4% |  | GPR, |  |
|  |  |  |  |  |  |  | GNR |  |
| 28 | 64 / M | 3.1×10^4^ | (-) | No growth | *Pseudomonas aeruginosa* |  | (-) | Oral bacteria |
|  |  |  |  |  | 73/87, 86.9% |  |  |  |
| 29 | 61 / M | <10^4^ | (-) | No growth | (-) |  | (-) | Oral bacteria |
| 30 | 81 / F | 5.0×10^6^ | GPC | *Streptococcus pneumoniae* | *Streptococcus pneumoniae* |  | N.A | |
|  |  |  |  |  | 71/79, 89.9% |  |  |  |
| 31 | 86 / M | 8.5×10^7^ | (-) | *Staphylococcus aureus* (MRSA) | *Corynebacterium simulans* |  | N.A | |
|  |  |  |  |  | 46/84, 54.8% |  |  |  |
| 32 | 91 / M | 1.6×10^7^ | (-) | *Staphylococcus* species | *Streptococcus oralis* |  | N.A | |
|  |  |  |  |  | 53/86, 61.6% |  |  |  |
| 33 | 68 / F | 2.5×10^6^ | (-) | *Corynebacterium* species 2+ | *Corynebacterium simulans* |  | N.A | |
|  |  |  |  |  | 77/77, 100% |  |  |  |
| 34 | 61 / M | 7.4×10^6^ | GPR, | *Haemophilus influenza* | *Haemophilus influenzae* |  | GPC, | No growth |
|  |  |  | GNR |  | 82/85, 96.5% |  | GPR, |  |
|  |  |  |  |  |  |  | GNR |  |
| 35 | 74 / M | 8.3×10^8^ | GPC, | *Staphylococcus aureus* (MRSA)*,*  *Pseudomonas aeruginosa* | *Streptococcus salivarius* |  | GPC, | *Staphylococcus aureus* (MRSA)*,*  *Pseudomonas aeruginosa* |
|  |  |  | GPR, |  | 34/79, 43.0% |  | GNR |  |
|  |  |  | GNR |  |  |  |  |  |
|  |  |  |  |  |  |  |  |  |
| 36 | 80 / F | 3.7×10^6^ | (-) | *Pseudomonas aeruginosa* | *Pseudomonas aeruginosa* |  | GPC, | *Pseudomonas aeruginosa* |
|  |  |  |  |  | 51/90, 56.7% |  | GPR |  |
| 37 | 71 / M | 6.2×10^5^ | GPC | No growth ^#^ | *Streptococcus pneumoniae* |  | GPC | *Streptococcus pneumoniae* |
|  |  |  |  |  | 74/85, 87.1% |  |  |  |
| 38 | 82 / F | 1.8×10^8^ | GPC | *Streptococcus pneumoniae*, | *Streptococcus pneumoniae* |  | GPC | *Streptococcus pneumoniae,*  *Staphylococcus aureus* (MRSA) |
|  |  |  |  |  | 83/83, 100% |  |  |  |
|  |  |  |  | *Staphylococcus aureus* (MSSA) |  |  |  |  |
| 39 | 80 / M | 1.7×10^7^ | GPC | *Staphylococcus aureus* (MRSA) | *Streptococcus salivarius* |  | GPC | *Staphylococcus aureus* (MRSA) |
|  |  |  |  |  | 62/76, 81.6% |  |  |  |
| 40 | 98 / F | 7.8×10^7^ | GPC, GNR | *Staphylococcus aureus* (MRSA) | *Streptococcus oralis* |  | GPC, | *Staphylococcus aureus* (MRSA) |
|  |  |  |  |  | 64/87, 73.6% |  | GNR |  |
| 41 | 41 / M | 1.2×10^7^ | GPR | No growth | *Haemophilus influenzae* |  | GPR, | No growth |
|  |  |  |  |  | 88/90, 97.8% |  | GNR |  |
| 42 | 70 / F | 3.7×10^6^ | GPC, | *Streptococcus pneumoniae*,  *Pseudomonas aeruginosa* | *Streptococcus pneumoniae* |  | GPR, | *Pseudomonas aeruginosa* |
|  |  |  | GPR, |  | 67/88, 76.1% |  | GNR |  |
|  |  |  | GNR |  |  |  |  |  |
|  |  |  |  |  |  |  |  |  |
| 43 | 89 / F | 2.4×10^6^ | GPR | *Pseudomonas aeruginosa* | *Haemophilus influenzae* |  | GPR | *Haemophilus influenza* |
|  |  |  |  |  | 92/94, 97.9% |  |  |  |
| 44 | 80 / F | 1.2×10^5^ | (-) | No growth | *Neisseria mucosa* |  | GPC, | *Staphylococcus aureus* (MRSA) |
|  |  |  |  |  | 33/60, 55.0% |  | GPR |  |
| 45 | 83 / M | 6.2×10^4^ | (-) | Oral bacteria | *Staphylococcus aureus* |  | GPC, | *Pseudomonas putida* |
|  |  |  |  |  | 26/85, 30.6% |  | GNR |  |
| 46 | 79 / M | 4.9×10^5^ | (-) | No growth | *Streptococcus intermedius* |  | GPC, | *Staphylococcus aureus* (MSSA)*,* |
|  |  |  |  |  | 75/75, 100% |  | GPR |  |
|  |  |  |  |  |  |  |  | *Pseudomonas aeruginosa* |
| 47 | 85 / M | 9.9×10^4^ | GNR | *Haemophilus influenza* | *Haemophilus influenzae* |  | GPR, | No growth |
|  |  |  |  |  | 56/56, 100% |  | GNR |  |
| 48 | 83 / M | 1.2×10^6^ | GPC, GNR | *Klebsiella pneumoniae*, | *Streptococcus oralis* |  | N.A | |
|  |  |  |  |  | 24/67, 35.8% |  |  |  |
|  |  |  |  | *Pseudomonas aeruginosa* |  |  |  |  |
| 49 | 91 / F | 5.1×10^7^ | GPC, | *Klebsiella pneumoniae* | *Klebsiella pneumoniae subsp.* |  | N.A | |
|  |  |  | GPR, |  |  |  |  |  |
|  |  |  | GNR |  | 45/77, 58.4% |  |  |  |
| 50 | 72 / M | 1.1×10^8^ | GNC, | *Haemophilus influenzae*, | *Haemophilus influenzae* |  | N.A | |
|  |  |  | GNR |  | 38/66, 57.6% |  |  |  |
|  |  |  |  | *Moraxella catarrhalis*, |  |  |  |  |
|  |  |  |  | *Pseudomonas aeruginosa* |  |  |  |  |
| 51 | 71 / M | 2.5×10^4^ | (-) | No growth | *Streptococcus oralis* |  | N.A | |
|  |  |  |  |  | 68/72, 94.4% |  |  |  |
| 52 | 85 / F | 6.2×10^5^ | GPC, | *Haemophilus influenza* | *Haemophilus influenzae* |  | N.A | |
|  |  |  | GNR |  | 86/86, 100% |  |  |  |
| 53 | 76 / M | 2.6×10^7^ | GNR | *Pseudomonas aeruginosa*,  *Enterobacter* species, *Citrobacter freundii* | *Neisseria* species |  | N.A | |
|  |  |  |  |  | 37/83, 44.6% |  |  |  |
|  |  |  |  |  |  |  |  |  |
|  |  |  |  |  |  |  |  |  |
| 54 | 85 / M | 9.2×10^5^ | (-) | Oral bacteria | *Enterobacter asburiae* |  | GPC, | *Enterobacter cloacae* |
|  |  |  |  |  | 21/89, 23.6% |  | GPR, |  |
|  |  |  |  |  |  |  | GNR |  |
| 55 | 74 / F | 6.2×10^5^ | GNR | *Haemophilus influenza* | *Haemophilus influenzae* |  | N.A | |
|  |  |  |  |  | 65/65, 100% |  |  |  |
| 56 | 86 / M | 5.0×10^5^ | GPC, | *Klebsiella pneumoniae* | *Streptococcus pseudopneumoniae* |  | GPC, | *Klebsiella pneumoniae* |
|  |  |  | GNR |  |  |  | GNR |  |
|  |  |  |  |  | 60/84, 71.4% |  |  |  |
| 57 | 82 / M | 4.1×10^7^ | GPC, | *Staphylococcus aureus*(MRSA), | *Streptococcus oralis* |  | N.A | |
|  |  |  | GNR |  | 53/75, 70.7% |  |  |  |
|  |  |  |  | *Streptococcus* species, |  |  |  |  |
|  |  |  |  | *Pseudomonas aeruginosa* |  |  |  |  |
| 58 | 62 / M | 3.1×10^4^ | (-) | No growth | *Prevotella* species |  | N.A | |
|  |  |  |  |  | 25/61, 41.0% |  |  |  |
| 59 | 69 / M | 9.3×10^5^ | GPC, | No growth | *Haemophilus influenzae* |  | GPC, | No growth |
|  |  |  | GPR |  | 44/80, 55.0% |  | GPR, |  |
|  |  |  |  |  |  |  | GNR |  |
| 60 | 76 / M | 9.1×10^7^ | GPC, | *Staphylococcus aureus* (MSSA) | *Staphylococcus aureus* |  | GPC, | *Staphylococcus aureus* (MRSA), |
|  |  |  | GPR |  | 52/95, 54.7% |  | GPR |  |
|  |  |  |  |  |  |  |  | *Pseudomonas aeruginosa* |
| 61 | 87 / F | 1.8×10^5^ | (-) | *Haemophilus influenzae* | *Haemophilus influenzae* |  | GPC, | *Haemophilus influenzae* |
|  |  |  |  |  | 80/81, 98.8% |  | GNR |  |
| 62 | 68 / M | 6.2×10^5^ | GPC, GNC | *Streptococcus pneumoniae* ^#^ | *Streptococcus pneumoniae* |  | N.A | |
|  |  |  |  |  | 75/87, 86.2% |  |  |  |
| 63 | 64 / M | 1.9×10^6^ | GPC, | *Staphylococcus aureus* (MRSA), | *Pseudomonas aeruginosa* |  | N.A | |
|  |  |  | GNR |  | 76/78, 97.4% |  |  |  |
|  |  |  |  | *Pseudomonas aeruginosa* |  |  |  |  |
| 64 | 74 / M | 1.7×10^7^ | GPC, | *Serratia marcescens* | *Staphylococcus aureus* |  | GPC, | No growth |
|  |  |  | GNR |  | 39/92, 42.4% |  | GPR |  |
| 65 | 72 / M | 1.2×10^5^ | GPC | *Streptococcus* species | *Fusobacterium canifelium* |  | GPC, | No growth |
|  |  |  |  |  | 46/87, 52.9% |  | GPR |  |
| 66 | 81 / F | 1.8×10^7^ | GPC | *Staphylococcus aureus* (MRSA),  *Streptococcus* species,  *Corynebacterium* species | *Streptococcus oralis* |  | GPC | *Staphylococcus aureus* (MRSA)*,* |
|  |  |  |  |  | 40/92, 43.5% |  |  |  |
|  |  |  |  |  |  |  |  | *Streptococcus species* |
|  |  |  |  |  |  |  |  |  |
| 67 | 85 / M | 6.0×10^7^ | GPC, GPR, GNR | *Staphylococcus aureus* (MRSA),  *Streptococcus* species,  *Klebsiella pneumoniae* | *Streptococcus intermedius* |  | GPC, | *Staphylococcus aureus* (MRSA), |
|  |  |  |  |  | 25/92, 27.2% |  | GPR, |  |
|  |  |  |  |  |  |  | GNC, |  |
|  |  |  |  |  |  |  | GNR | *Streptococcus* species |
|  |  |  |  |  |  |  |  |  |
| 68 | 68 / M | 3.1×10^4^ | (-) | No growth | *Pseudomonas aeruginosa* |  | (-) | No growth |
|  |  |  |  |  | 39/70, 55.7% |  |  |  |
| 69 | 79 / F | 6.2×10^5^ | (-) | No growth | *Streptococcus salivarius* |  | GPC, | *Staphylococcus aureus* (MSSA), |
|  |  |  |  |  | 27/62, 43.5% |  | GPR, |  |
|  |  |  |  |  |  |  | GNR |  |
|  |  |  |  |  |  |  |  | *Escherichia coli* |
| 70 | 85 / M | 4.8×10^7^ | GPC, | Oral bacteria | *Veillonella dispar* |  | GPC, | *Streptococcus mitis* |
|  |  |  | GPR, |  | 35/62, 56.5% |  | GPR, |  |
|  |  |  | GNR |  |  |  | GNR |  |
| 71 | 77 / M | 9.3×10^5^ | GPC | No growth | *Haemophilus influenzae* |  | N.A | |
|  |  |  |  |  | 56/85, 65.9% |  |  |  |
| 72 | 74 / M | 3.1×10^4^ | (-) | *Haemophilus influenza* | *Haemophilus influenzae* |  | GNR | *Haemophilus influenza* |
|  |  |  |  |  | 80/86, 93.0% |  |  |  |
| 73 | 82 / F | 9.3×10^7^ | GPC, | No growth | *Gemella haemolysans* |  | N.A | |
|  |  |  | GPR, |  | 37/68, 54.4% |  |  |  |
|  |  |  | GNR |  |  |  |  |  |
| 74 | 65 / F | 9.3×10^5^ | GNR | *Pseudomonas aeruginosa* | *Pseudomonas aeruginosa* |  | N.A | |
|  |  |  |  |  | 81/81, 100% |  |  |  |
| 75 | 94 / F | 4.3×10^7^ | GPC, | *Klebsiella pneumoniae*, | *Klebsiella* species |  | GPC, | *Klebsiella pneumoniae* |
|  |  |  | GPR, |  | 39/85, 45.9% |  | GPR, |  |
|  |  |  | GNR | *Escherichia coli*, |  |  | GNR |  |
|  |  |  |  | *Acinetobacter* species |  |  |  |  |
| 76 | 83 / M | 9.9×10^6^ | GPC | *Streptococcus anginosus* species | *Streptococcus intermedius* |  | GPC, | *Streptococcus anginosus* species, |
|  |  |  |  |  | 70/74, 94.6% |  | GNR |  |
|  |  |  |  |  |  |  |  | *Klebsiella pneumoniae* |
| 77 | 93 / M | 1.6×10^7^ | GPC, | *Staphylococcus aureus*(MRSA), | *Corynebacterium simulans* |  | N.A | |
|  |  |  | GPR |  | 53/90, 58.9% |  |  |  |
|  |  |  |  | *Corynebacterium* species |  |  |  |  |
| 78 | 68 / M | 6.2×10^6^ | GPC, GPR, | *Pseudomonas aeruginosa*, | *Corynebacterium striatum* |  | N.A | |
|  |  |  |  |  | 49/68, 72.1% |  |  |  |
|  |  |  | GNR | *Klebsiella pneumoniae* |  |  |  |  |
| 79 | 75 / M | 1.6×10^7^ | GPC, GPR, | *Staphylococcus aureus*, | *Porphyromonas gluae* |  | GPC, | *Staphylococcus aureus* |
|  |  |  |  |  | 44/79, 55.7% |  | GPR, |  |
|  |  |  | GNR | *Streptococcus* species, |  |  | GNR |  |
|  |  |  |  | *Corynebacterium* species |  |  |  |  |
| 80 | 69 / M | 1.2×10^5^ | (-) | *Streptococcus pneumoniae* | *Streptococcus pneumoniae* |  | GPC, | *Klebsiella oxytoca* |
|  |  |  |  |  | 81/94, 86.2% |  | GNR |  |
| 81 | 69 / F | 5.0×10^6^ | GPC, GNR | *Streptococcus* species, | *Streptococcus oralis* |  | GPC, | *Streptococcus* species, |
|  |  |  |  |  | 60/91, 65.9% |  | GNR |  |
|  |  |  |  | *Klebsiella pneumoniae* |  |  |  | *Klebsiella pneumoniae* |
| 82 | 87 / M | 3.7×10^6^ | (-) | No growth | *Klebsiella* species |  | GNR | *Klebsiella pneumoniae* |
|  |  |  |  |  | 26/81, 32.1% |  |  |  |
| *Definition of abbreviation*: BALF, bronchoalveolar lavage fluid; GPC, gram positive cocci; GPR, gram positive rods; GNC, gram negative cocci; GNR, gram negative rods; N.A, not analyzed | | | | | | | | |
| ^§1^A phylotype sharing 97% or higher homology with the sequence of the type strain was assumed to be a presumptive species, and a phylotype with a sequence sharing between 90% and 97% of the type strain was assumed to be a presumptive genus. | | | | | | | | |
| ^§2^Serological tests of *Mycoplasma pneumoniae* and *Chlamidophila pneumoniae* were performed in 18 patients and the positive results were none, respectively. | | | | | | | | |
| ^§3^Serological tests of urinary antigen tests of *Streptococcus pneumoniae* and *Legionella pneumophila* were performed in 51 patients and the positive results were 4 and 0, respectively. | | | | | | | | |
| ^#^The result of urinary antigen tests to detect *Streptococcus pneumoniae* was positive. | | | | | | | | |
