# Supplementary material for: Bacteriological Assessment of Healthcare-Associated Pneumonia Using a Clone Library Analysis
Source: PLoS One. 2015 Apr 15;10(4):e0124697. doi: 10.1371/journal.pone.0124697 (PMC4398420; doi:10.1371/journal.pone.0124697)
Supplement: S2 Table — (DOCX) [file pone.0124697.s002.docx]

S2 Table. Results of the molecular method and antibiotics efficacy in patients with positive cultivation of *Pseudomonas aeruginosa*

| No.^§^ | Cultivation | |  | The results of Clone Library Method of 16S ribosomal RNA gene | |  | Effective antibiotics |
| --- | --- | --- | --- | --- | --- | --- | --- |
|  | Sputum | BALF |  | BALF | |  |  |
|  |  |  |  | Predominant phylotype (%, Clones/clones) | Proportion of *P. aeruginosa (%, Clones/clones)* |  |  |
| 1 | *P. aeruginosa* | *P. aeruginosa* |  | *P. aeruginosa* |  |  | CPFX |
|  |  |  |  | 100% (77/77) |  |  |  |
| 2 | *P. aeruginosa* | *P. aeruginosa* |  | *S. aureus* |  |  | TAZ/PIPC |
|  | MSSA | MSSA |  | 95.4% (83/87) | 0% (0/87) |  |  |
| 3 | *P. aeruginosa* | *P. aeruginosa* |  | *P. aeruginosa* |  |  | MEPM |
|  |  |  |  | 23.4% (15/64) |  |  |  |
| 4 | *P. aeruginosa* | *P. aeruginosa* |  | *S. pneumoniae* |  |  | TAZ/PIPC |
|  |  | *S. pneumoniae* |  | 76.1% (67/88) | 23.1% (21/88) |  |  |
| 5 | *P. aeruginosa* | *P. aeruginosa* |  | *S. salivarius* |  |  | MEPM→TAZ/PIPC+VCM |
|  | MRSA | MRSA |  | 43.0% (34/79) | 0% (0/79) |  |  |
| 6 | *P. aeruginosa* | *P. aeruginosa* |  | *P. aeruginosa* |  |  | IPM/CS |
|  |  |  |  | 56.7% (51/90) |  |  |  |
| 7 | *P. aeruginosa* | No growth |  | *S. intermedius* |  |  | MEPM |
|  | MSSA |  |  | 0% (75/75) | 0% (0/75) |  |  |
| 8 | N.A | *P. aeruginosa* |  | *S. oralis* |  |  | SBT/ABPC |
|  |  | MRSA |  | 70.7% (53/75) | 6.7% (5/75) |  |  |
|  |  | *Streptococcus* species |  |  |  |  |  |
| 9 | *P. aeruginosa* | MSSA |  | *S. aureus* |  |  | TAZ/PIPC |
|  | MRSA |  |  | 54.7% (52/95) | 0% (0/95) |  |  |
| 10 | *H. influenzae* | *P. aeruginosa* |  | *H. influenzae* |  |  |  |
|  |  |  |  | 97.9% (92/94) | 0% (0/94) |  | MEPM |
| 11 | N.A | *P. aeruginosa* |  | *S. oralis* |  |  | LVFX |
|  |  | *K. pneumoniae* |  | 35.8% (24/67) | 0% (0/67) |  |  |
| 12 | N.A | *P. aeruginosa* |  | *H. influenzae* |  |  |  |
|  |  | *H. influenzae* |  | 57.6% (38/66) | 0% (0/94) |  |  |
|  |  | *M.catarhalis* |  |  |  |  |  |
| 13 | N.A | *P. aeruginosa* |  | *Neisseria* species |  |  | LVFX |
|  |  | *Enterobacter* species |  | 44.6% (37/83) | 3.6% (3/83) |  |  |
|  |  | *Citrobacter freundii* |  |  |  |  |  |
| 14 | N.A | *P. aeruginosa* |  | *P. aeruginosa* |  |  | TAZ/PIPC |
|  |  | MRSA |  | 97.4% (76/78) |  |  |  |
| 15 | N.A | *P. aeruginosa* |  | *P. aeruginosa* |  |  | TAZ/PIPC |
|  |  |  |  | 100% (81/81) |  |  |  |
| 16 | N.A | *P. aeruginosa* |  | *Corynebacterium striatum* |  |  | TAZ/PIPC |
|  |  | *K. pneumoniae* |  | 72.1% (49/68) | 2.9% (2/68) |  |  |
| *Definition of abbreviation*: MRSA, methicillin-resistant *staphylococcus aureus*; MSSA, methicillin-susceptible *staphylococcus aureus*; BALF, bronchoalveolar lavage fluid; SBT/ABPC, ampicillin/sulbactam; TAZ/PIPC, piperacillin/tazobactam; MEPM, meropenem; LVFX, levofloxacin; CPFX, ciprofloxacin; IPM/CS, imipenem/cilastatin; VCM, vancomycin; N.A, not analyzed | | | | | | | |
| ^§^Case numbers were as follow: No.1, case3; No.2, case21; No.3, case27; No.4, case42; No.5, case35; No.6, case36; No.7, case46; No.8, case57; No.9, case60; No.10, case43; No.11, case48; No.12, case50; No.13, case53; No.14, case63; No.15, case74; No.16, case78 | | | | | | | |
