# Supplementary material for: Bacteriological Assessment of Healthcare-Associated Pneumonia Using a Clone Library Analysis
Source: PLoS One. 2015 Apr 15;10(4):e0124697. doi: 10.1371/journal.pone.0124697 (PMC4398420; doi:10.1371/journal.pone.0124697)
Supplement: S3 Table — (DOCX) [file pone.0124697.s003.docx]

S3 Table. Results of the molecular method and antibiotics efficacy in patients with positive cultivation of *Klebsiella pneumoniae*

| No.^§^ | Cultivation | |  | The results of Clone Library Method of 16S ribosomal RNA gene | |  | Effective antibiotics |
| --- | --- | --- | --- | --- | --- | --- | --- |
|  | Sputum | BALF |  | BALF | |  |  |
|  |  |  |  | Predominant phylotype (%, Clones/clones) | Proportion of *K. pneumoniae(%, Clones/clones)* |  |  |
| 1 | *K. pneumoniae* | *K. pneumoniae* |  | *S. pseudopneumoniae* |  |  | MEPM |
|  |  |  |  | 71.4% (60/84) | 0% (0/84) |  |  |
| 2 | *K. pneumoniae* | *K. pneumoniae* |  | *Klebsiella* species |  |  | MEPM |
|  |  | *E. coli* |  | 45.9% (39/85) |  |  |  |
|  |  | *Acinetobacter* species |  |  |  |  |  |
| 3 | *K. pneumoniae* | *S. anginosus* species |  | *S. intermedius* | |  | AZM |
|  | *S. anginosus* species |  |  | 94.6% (70/74) | 0% (0/74) |  |  |
| 4 | *K. pneumoniae* | *K. pneumoniae* |  | *S. oralis* |  |  | MEPM |
|  | *Streptococcus* species | *Streptococcus* species |  | 65.9% (60/91) | 1.1% (1/91) |  |  |
| 5 | *K. pneumoniae* | No growth |  | *Klebsiella species* |  |  | TAZ/PIPC |
|  |  |  |  | 32.1% (26/81) |  |  |  |
| 6 | *K. oxytoca* | *S. pneumoniae* |  | *S. pneumoniae* |  |  | SBT/ABPC |
|  |  |  |  | 86.2% (81/94) | 0% (0/94) |  |  |
| 7 | N.A | *K. pneumoniae* |  | *S. oralis* |  |  | LVFX |
|  |  | *P. aeruginosa* |  | 35.8% (24/67) | 9.0% (6/67) |  |  |
| 8 | N.A | *K. pneumoniae* |  | *K. pneumoniae* |  |  | MEPM |
|  |  |  |  | 58.4% (45/77) |  |  |  |
| 9 | MRSA | *K. pneumoniae* |  | *S. oralis* |  |  | LVFX |
|  | *Streptococcus* species | MRSA |  | 43.5% (40/92) | 0% (0/92) |  |  |
|  |  | *Streptococcus* species |  |  |  |  |  |
|  |  | *Corynebacterium* species |  |  |  |  |  |
| 10 | N.A | *K. pneumoniae* |  | *Corynebacterium striatum* |  |  | TAZ/PIPC |
|  |  | *P. aeruginosa* |  | 72.1% (49/68) | 8.8% ( 6/68) |  |  |
| *Definition of abbreviation*: MRSA, methicillin-resistant *staphylococcus aureus*; MSSA, methicillin-susceptible *staphylococcus aureus*; BALF, bronchoalveolar lavage fluid; SBT/ABPC, ampicillin/sulbactam; TAZ/PIPC, piperacillin/tazobactam; MEPM, meropenem; LVFX, levofloxacin; AZM, azithromycin; N.A, not analyzed | | | | | | | |
| ^§^Case numbers were as follow: No.1, case56; No.2, case75; No.3, case76; No.4, case81; No.5, case82; No.6, case80; No.7, case48; No.8, case49; No.9, case66; No.10, case78 | | | | | | | |
