# Supplementary material for: Bacteriological Assessment of Healthcare-Associated Pneumonia Using a Clone Library Analysis
Source: PLoS One. 2015 Apr 15;10(4):e0124697. doi: 10.1371/journal.pone.0124697 (PMC4398420; doi:10.1371/journal.pone.0124697)

S1 Figure. Percentage of detected phylotypes in the “monobacteria-dominant” and “mixed bacteria” groups.

A) Percentage of phylotypes in each sample among the 30 patients in the “monobacteria-dominant group”; B) Percentage of phylotypes in each sample among the 50 patients in the “mixed bacteria group.” Phylotypes present at a rate of less than 5% in each library were classified as “others.”


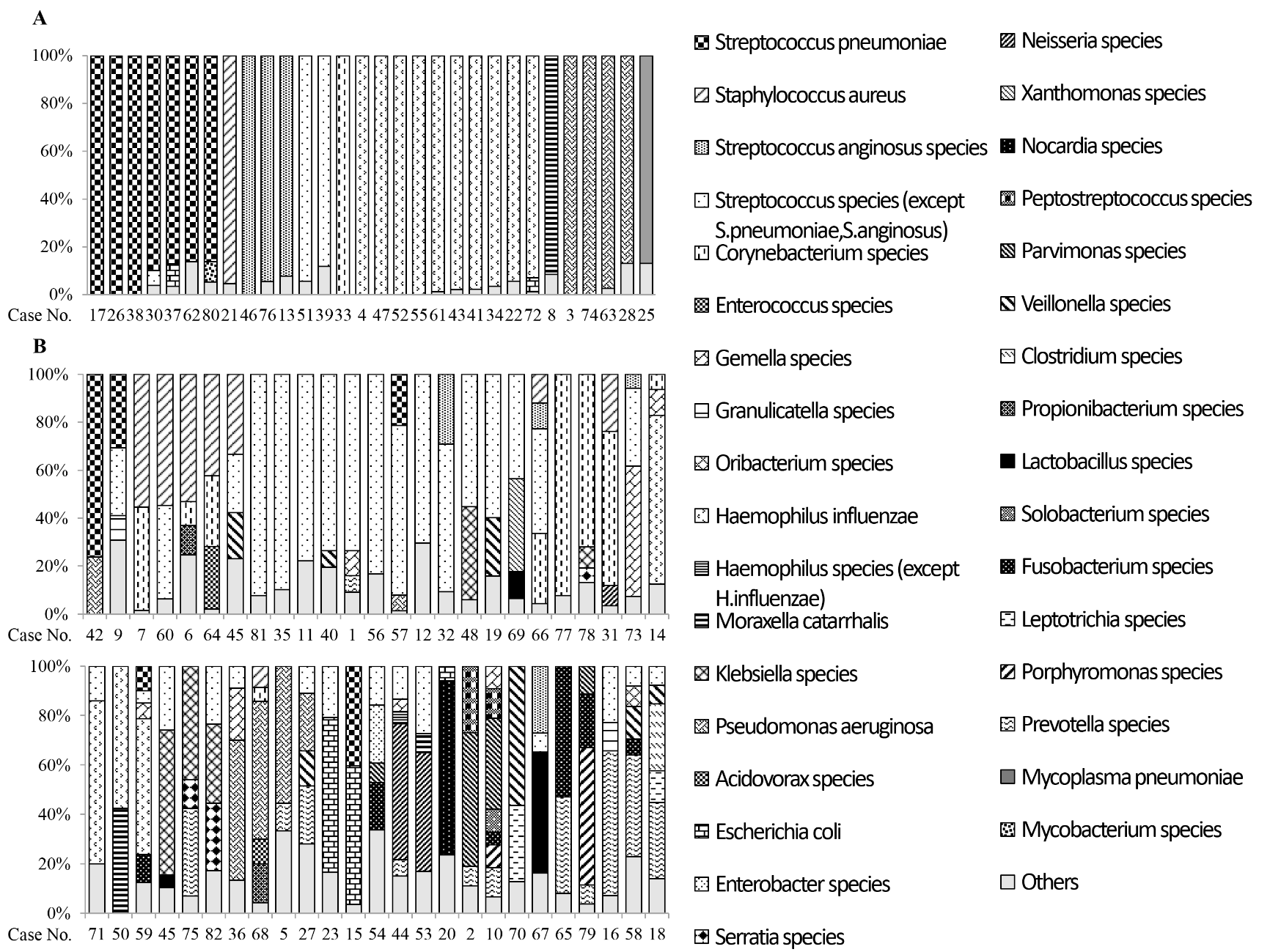

Supplement: S1 Fig — A) Percentage of phylotypes in each sample among the 30 patients in the “monobacteria-dominant group”; B) Percentage of phylotypes in each sample among the 50 patients in the “mixed bacteria group.” Phylotypes present at a rate of less than 5% in each library were classified as “others.” (DOCX) [file pone.0124697.s004.docx]
